# Erratum: receptor-like kinase complexes in plant innate immunity

**DOI:** 10.3389/fpls.2012.00264

**Published:** 2012-11-28

**Authors:** Christiaan Greeff, Milena Roux, John Mundy, Morten Petersen

**Affiliations:** Department of Biology, Copenhagen UniversityCopenhagen, Denmark

**A commentary on**

**Receptor-like kinase complexes in plant innate immunity**

Greeff et al. (2012). Front. Plant Sci. 3:209. doi: 10.3389/fpls.2012.00209

## In Figure 1

After our article was published online, it was brought to our attention that Figure 1B could give the impression that the current model is that FLS2 and BIR1 are found together in complexes with BAK1 upon flg22 perception. Since this is not the case and we do not discuss this possibility in the text we have decided to omit BIR1 from Figure 1B. It also appears that all the receptors shown in Figure 1D share all the outputs upon infection, however, it should be noted that all these responses have not yet been demonstrated for Xa21. We have thus changed the text embedded in the figure to reflect this. In addition we showed in Figures 1B and 1C that FLS and EFR activation results in phosphorylation of BAK1. This, however, is only an effect of receptor activation and is misleading as it stands, therefore have changed the arrow heads. Finally, the yellow block arrow in Figure 1A indicating dephosphorylation has been changed to black to make the figure clearer. Figure 1 legend should then accordingly read “Black blunt arrows indicate dephosphorylation of a substrate protein.”

**Figure 1:**
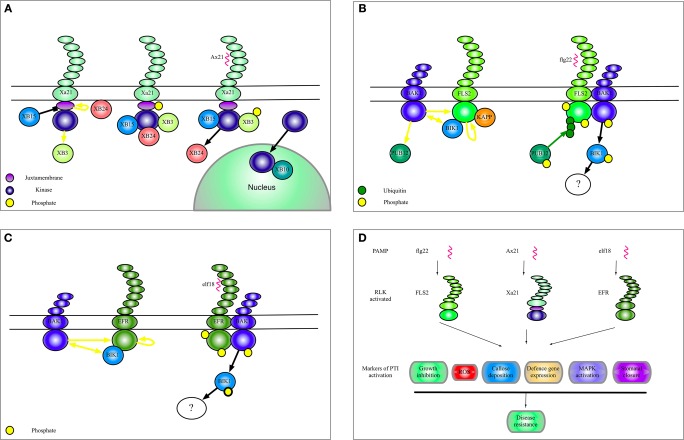
**Complexes of Xa21, FLS2, and EFR. (A)** A model to summarize the current data regarding Xa21 function as discussed in this manuscript. **(B)** Illustration of the complexes formed by the RLK FLS2. The yellow dots indicate phosphorylation of a protein. Yellow arrows indicate phosphorylation of a substrate protein. Black blunt arrows indicate dephosphorylation of a substrate protein. Green dots and green arrows indicate ubiquitination. Black arrows indicate translocation, association, or dissociation. **(C)** Selected interactors of the RLK EFR. **(D)** Shows biological effects of selected RLK activation.

## In text

We would further like to change the following mistake in the text where we write “BAK1 and FLS2 phosphorylate BIK1 (Lu et al., 2010) and BIK1 in turn phosphorylates both FLS2 and BAK1” (page 3, bottom of left column). However, this is incorrect, instead the sentence should read “BAK1 phosphorylate BIK1, and BIK1 phosphorylates BAK1 (Lu et al., 2010).”
